# Supplementary material for: Interfacial dynamics mediate surface binding events on supramolecular nanostructures
Source: Nat Commun. 2024 Sep 5;15:7749. doi: 10.1038/s41467-024-51494-4 (PMC11377763; doi:10.1038/s41467-024-51494-4)
Supplement: Supplementary file 1 — Supplementary Information [file 41467_2024_51494_MOESM1_ESM.pdf]

Supplementary Information for

Interfacial dynamics mediate surface binding events on supramolecular nanostructures

Ty Christoff-Tempesta[1,†,6], Yukio Cho[1,†,7], Samuel J. Kaser[2], Linnaea D. Uliassi[1], Xiaobing Zuo[3], Shayna L. Hilburg[4], Lilo D. Pozzo[4], Julia H. Ortony[1,5,*]

[1] Department of Materials Science and Engineering, Massachusetts Institute of Technology, Cambridge, MA 02139, USA

[2] Department of Chemistry, Massachusetts Institute of Technology, Cambridge, MA 02139, USA

[3] X-ray Science Division, Advanced Photon Source, Argonne National Laboratory, Lemont, IL 60439, USA

[4] Department of Chemical Engineering, University of Washington, Seattle, WA 98195, USA

[5] Department of Chemistry and Biochemistry, University of California San Diego, La Jolla, CA 92093, USA

[6] Present address: Department of Chemical and Biomolecular Engineering, University of Delaware, Newark, DE 19716, USA

[7] Present address: SLAC National Accelerator Laboratory, Stanford University, Menlo Park, CA 94025, USA

[†] These authors contributed equally to this work.

* Correspondence to: jortony@ucsd.edu

Materials

Methyl 4-aminobenzoate (Sigma Aldrich, 98%), 3,3-dimethylbutyric acid (Sigma Aldrich, 98%), *N*-Boc-*p*-phenylenediamine (BPP, Sigma Aldrich, 97%), *N*-Boc-3-[2-(2-aminoethoxy)ethoxy]propionic acid (Ambeed Inc., 95%), Boc-15-amino-4,7,10,13-tetraoxapentadecanoic acid (Chem Impex. 95%), 2-(4,7,10-tris(2-tert-butoxy-2-oxoethyl)-1,4,7,10-tetraazacyclododecan-1-yl)acetic acid (DOTA-tris(*t*-Bu ester), AstaTech, 95%), 4-carboxy-2,2,6,6-tetramethylpiperidine 1-oxyl (4-carboxy-TEMPO, Sigma Aldrich, 97%), 1-ethyl-3-(3-dimethylaminopropyl)carbodiimide hydrochloride (EDC, TCI Chemicals, 98%), *N*,*N*'-Diisopropylcarbodiimide (DIC, Chem Impex. 99%), 4-dimethylaminopyridine (DMAP, TCI Chemicals, 99%), ethyl cyano(hydroxyimino)acetate (TCI Chemicals, 98%), lithium hydroxide monohydrate ($LiOH \cdot H_2O$, Alfa Aesar, 98%), sodium bicarbonate ($NaHCO_3$, Alfa Aesar, 99%), hydrochloric acid (HCl, Alfa Aesar, 36%), sodium sulfate (Na_2SO_4, Fisher Scientific, 99%), magnesium sulfate ($MgSO_4$, J.T. Baker, anhydrous, 99%), sodium chloride (HCl, Fisher Scientific, 99%), trifluoroacetic acid (TFA, Alfa Aesar, 99%), methanol (Fisher Scientific), acetonitrile (Fisher Scientific), methylene chloride (Fisher Scientific), *N,N*-dimethylformamide (dimethylformamide, Fisher Scientific), and ethyl acetate (Fisher Scientific) were used as received without further purification.

Synthesis of aramid amphiphiles

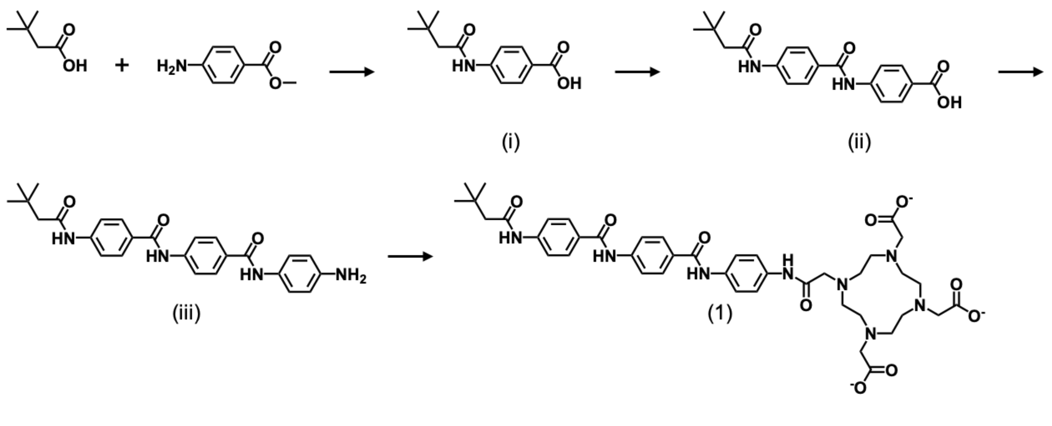

Supplementary Figure 1. Synthesis scheme to obtain compound (**1**)

4-(3,3-dimethylbutanamido)benzoic acid (**i**): A solution of 3,3-dimethylbutyric acid (50 mmol), methyl 4-aminobenzoate (33 mmol), EDC (100 mmol), and DMAP (100 mmol) in dimethylformamide (150 mL) was stirred at 60 °C for 24 h. After the reaction, excess deionized water was added to the solution to obtain a precipitate, which was collected by filtration. The crude precipitate was further mixed with methanol and then precipitated in a 5 wt% sodium bicarbonate (aq) solution. The precipitate was then obtained by filtration, washed with 5 wt% sodium bicarbonate (aq) solution, and dried under vacuum. Lithium hydroxide monohydrate (290 mmol) was dissolved in deionized water (60 mL), and added to a stirred solution of the precipitate compound in tetrahydrofuran (240 mL) and methanol (120 mL). The mixture was refluxed for 24 h. The volatile fraction was then evaporated under reduced pressure and neutralized with an aqueous 1% hydrochloric acid solution. The precipitate was filtered off, washed with water, and dried under vacuum to afford the product (yield: 89.4%).

4-(4-(3,3-dimethylbutanamido)benzamido)benzoic acid (**ii**): A solution of methyl 4-aminobenzoate (45 mmol), compound **i** (30 mmol), EDC (90 mmol), and DMAP (90 mmol) in dimethylformamide (200 mL) was stirred at 60 °C for 24 h. After the reaction, excess deionized

water was added to the solution to obtain a precipitate, which was collected by filtration. The crude precipitate was further washed with methanol and acetonitrile, and then dried under vacuum. Lithium hydroxide monohydrate (300 mmol) was dissolved in deionized water (80 mL) and added to a stirred solution of the precipitate compound in tetrahydrofuran (320 mL) and methanol (160 mL). The mixture was refluxed for 24 h. The volatile fraction was then evaporated under reduced pressure and neutralized with an aqueous 1% hydrochloric acid solution. The precipitate was filtered off, washed with water and methanol, and dried under vacuum to afford the product (yield: 62.0%).

N-(4-(amino)phenyl)-4-(4-(3,3-dimethylbutanamido)benzamido)benzamide (**iii**): A solution of BPP (36 mmol), compound **ii** (18 mmol), EDC (54 mmol), and DMAP (54 mmol) in dimethylformamide (200 mL) was stirred at 60 °C for 24 h. After the reaction, excess deionized water was added to the solution to obtain a precipitate, which was collected by filtration. The crude precipitate was further washed with methanol, and then dried under vacuum. TFA (20 mL) and the precipitate compound were mixed in chloroform (180 mL) for 24 h at room temperature. Then, the volatile components were removed *in vacuo* and the remaining mixture was washed with saturated sodium bicarbonate (aq) solution to afford the final product, which was obtained by filtration and dried under vacuum (yield: 91.4%).

2,2',2''-(10-(2-((4-(4-(4-(3,3-dimethylbutanamido)benzamido)benzamido)phenyl)amino)-2-oxoethyl)-1,4,7,10-tetraazacyclododecane-1,4,7-triyl)triacetate (**1**): A solution of DOTA-tris(*t*-Bu ester) (1.65 mmol), compound **ii** (1.38 mmol), EDC (4.13 mmol), and DMAP (4.13 mmol) in dimethylformamide (25 mL) was stirred at 60 °C for 24 h. After the reaction, the solvent was removed *in vacuo* and the mixture was suspended in a solution of 20 g sodium chloride dissolved in 100 mL deionized water to obtain a precipitate. The precipitate was obtained by

filtration and dried under vacuum. The precipitate was then mixed with TFA (3 mL) and stirred for 24 h at room temperature. Afterwards, the volatile components were removed *in vacuo* and the remaining residue was mixed with ethyl acetate to afford the final product as a precipitate. The product was obtained by filtration and dried under vacuum (yield: 43.2%). NMR (400 MHz, Bruker Avance III DPX 400, DMSO-*d*): 7.95 (m, 8H), 7.75 (t, 4H), 7.57 (d, 2H), 4.04 (m, 1H), 3.87 (s, 6H), 3.74 (s, 3H), 3.64 (s, 1H), 2.7 (m, 4H) , 2.25 (s, 2H), 2.00 (s, 1H), 1.90 (s, 1H), 1.17 (m, 2H), 1.05 (s, 9H) ppm. MS (MALDI-ToF) $[M + H]^+$ m/z calculated: 831.40; $[M + H]^+$ found: 831.42.

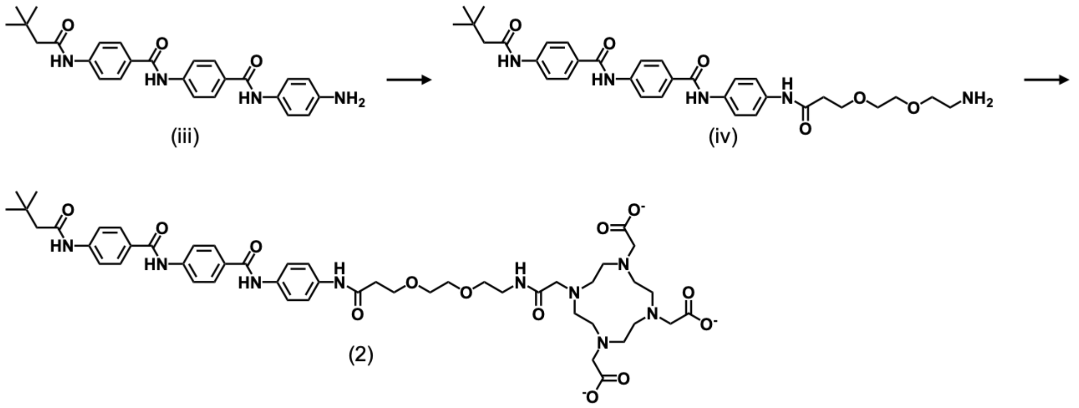

Supplementary Figure 2. Synthesis scheme to obtain compound (**2**)

N-(4-(3-(2-(2-aminoethoxy)ethoxy)propanamido)phenyl)-4-(4-(3,3dimethylbutanamido)benzamido)benzamide (**iv**): A solution of compound **iii** (0.90 mmol), *N*-Boc-3-[2-(2-aminoethoxy)ethoxy]propionic acid (1.80 mmol), DIC (3.60 mmol), and ethyl cyano(hydroxyimino)acetate (3.60 mmol) in a mixture of dimethylformamide (50 mL) and methylene chloride (50 mL) was stirred at room temperature for 24 h. After the reaction, the solvent was removed *in vacuo*, and the remaining residue was washed with deionized water and acetonitrile. The isolated compound was then reacted with TFA (10 mL) in methylene

chloride (100 mL) at room temperature for 9 h. The volatile fraction was evaporated under reduced pressure. A cosolvent of 9 : 1 diethyl ether : methylene chloride was added to recrystallize the product, which was subsequently separated from the solvent by centrifugation at 4000 rpm for 3 minutes. Afterwards, the precipitate was collected. The remaining product in the centrifuge tube was resuspended in solvent and centrifuged to collect three more times. The solid precipitate was collected and dried under vacuum (yield: 97.6%).

2,2',2"-(10-(2-((2-(2-(3-((4-(4-(4-(3,3dimethylbutanamido)benzamido)benzamido)phenyl)amino)-3-oxopropoxy)ethoxy)ethyl)amino)-2-oxoethyl)-1,4,7,10-tetraazacyclododecane-1,4,7-triyl)triacetate (**2**): A solution of compound **iv** (0.18 mmol), DOTA-tris(*t*-Bu ester) (0.36 mmol), DIC (0.54 mmol), and ethyl cyano(hydroxyimino)acetate (0.54 mmol) in a mixture of dimethylformamide (10 mL) and methylene chloride (10 mL) was stirred at room temperature for 72 h. After the reaction, the solvent was removed *in vacuo* and the remaining residue was dissolved in methylene chloride. The solution was washed by a cosolvent of 5:5 deionized water:methanol via solvent extraction and the organic fraction in methylene chloride was dried using magnesium sulfate and filtered. The organic layer was retained and the volatile fraction was evaporated under reduced pressure. A cosolvent of 9 : 1 diethyl ether : methylene chloride was added to recrystallize the product, which was subsequently separated from the solvent by centrifugation at 4000 rpm for 3 minutes. Afterwards, the precipitate was collected. The remaining compound in the centrifuge tube was resuspended in solvent and centrifuged to collect three more times. All retained compound was then dried in vacuum. The isolated compound was then reacted with TFA (4 mL) in methylene chloride (10 mL) at room temperature for 24 h. The volatile fraction was evaporated under reduced pressure, and the remaining residue was suspended in ethyl acetate. This mixture was centrifuged at 4000 rpm for 3 minutes and the precipitate was collected. The remaining product in the centrifuge tube

was resuspended in ethyl acetate and centrifuged to collect three more times. All retained product was then dried under vacuum (yield: 24.9%). NMR (400 MHz, Bruker Avance III DPX 400, DMSO-*d*): 7.96 (6H, m), 7.76 (2H, d), 7.70 (2H, d), 7.57 (2H, d), 3.72 (2H, m), 3.62 (2H, s), 3.53 (6H, t), 3.45 (2H, t), 3.25 (2H, m), 3.00 (10H, t), 2.56 (2H, dd), 2.25 (2H, s), 1.05 (9H, s) ppm. MS (MALDI-ToF) $[M + H]^+$ m/z calculated: 990.49; $[M + H]^+$ found: 990.54.

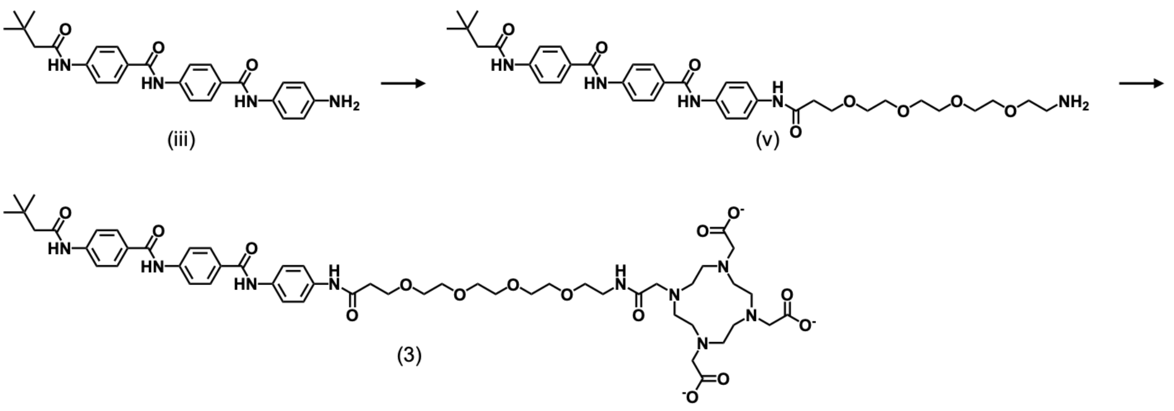

Supplementary Figure 3. Synthesis scheme to obtain compound (**3**)

N-(4-(1-amino-3,6,9,12-tetraoxapentadecan-15-amido)phenyl)-4-(4-(3,3-dimethylbutanamido)benzamido)benzamide (**v**): A solution of compound **iii** (0.69 mmol), Boc-15-amino-4,7,10,13-tetraoxapentadecanoic acid (1.37 mmol), DIC (1.37 mmol), and ethyl cyano(hydroxyimino)acetate (1.37 mmol) in a mixture of dimethylformamide (35 mL) and methylene chloride (35 mL) was stirred at room temperature for 24 h. After the reaction, the solvent was removed *in vacuo*, and the remaining residue was washed with deionized water. The isolated crude product was then reacted with TFA (10 mL) in methylene chloride (100 mL) at room temperature for 5 h. The volatile fraction was evaporated under reduced pressure. A cosolvent of 9 : 1 diethyl ether : methylene chloride was added to recrystallize the product, which was subsequently separated from the solvent by centrifugation at 4000 rpm for 3 minutes. Afterwards, the precipitate was collected. The remaining product in the centrifuge tube was

resuspended in solvent and centrifuged to collect three more times. The solid precipitate was collected and dried in vacuum (yield: 77.3%).

2,2',2''-(10-(18-((4-(4-(4-(3,3-dimethylbutanamido)benzamido)benzamido)phenyl)amino) -2,18-dioxo-6,9,12,15-tetraoxa-3-azaoctadecyl)-1,4,7,10-tetraazacyclododecane-1,4,7-triyl)triacetate (**3**): A solution of compound **v** (0.28 mmol), DOTA-tris(*t*-Bu ester) (0.56 mmol), DIC (0.84 mmol), and ethyl cyano(hydroxyimino)acetate (0.84 mmol) in a mixture of dimethylformamide (16 mL) and methylene chloride (16 mL) was stirred at room temperature for 72 h. After the reaction, the solvent was removed *in vacuo* and the remaining residue was dissolved in methylene chloride. The solution was washed by a cosolvent of 5 : 5 deionized water : methanol via solvent extraction and the organic fraction in methylene chloride was dried using magnesium sulfate and filtered. The organic layer was retained and the volatile fraction was evaporated under reduced pressure. A cosolvent of 9 : 1 diethyl ether : methylene chloride was added to recrystallize the product, which was separated from the solvent by centrifugation at 4000 rpm for 3 minutes. Afterwards, the precipitate was collected. The remaining compound in the centrifuge tube was resuspended in solvent and centrifuged to collect three more times. All retained compound was then dried in vacuum. The isolated compound was then reacted with TFA (8 mL) in methylene chloride (20 mL) at room temperature for 24 h. The volatile fraction was evaporated under reduced pressure. The remaining residue was suspended in ethyl acetate and collected by centrifugation at 4000 rpm for 3 minutes and the precipitate was collected. The remaining product in the centrifuge tube was resuspended in solvent and centrifuged to collect three more times. All retained product was then dried under vacuum. (yield: 44.5%). NMR (400 MHz, Bruker Avance III DPX 400, DMSO-*d*): 7.96 (6H, m), 7.76 (2H, d), 7.70 (2H, d), 7.57 (2H, d), 3.71 (2H, t), 3.62 (4H, s),

3.53 (14H, t), 3.40 (4H, t), 3.00 (16H, t), 3.30 (4H, d), 2.25 (2H, s), 1.05 (9H, s) ppm. MS (MALDI-ToF) $[M + H]^+$ m/z calculated: 1100.54; $[M + Na]^+$ found: 1100.53.

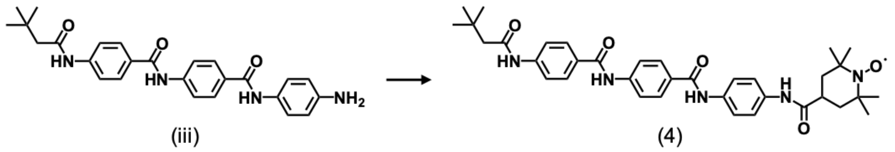

Supplementary Figure 4 | Synthesis scheme to obtain compound (**4**)

N-(4-(4-(4-(3,3-dimethylbutanamido)benzamido)benzamido)phenyl)-1-hydroxy-2,2,6,6 -tetramethylpiperidine-4-carboxamide (**4**): A solution of compound (**iii**) (0.34 mmol), 4 Carboxy-TEMPO (1.02 mmol), EDC (1.02 mmol), DMAP (1.02 mmol), and DIPEA (3.04 mmol) in 7 mL dimethylformamide and 7 mL methylene chloride was mixed for 24 h at room temperature. The solution was then rotovapped to remove volatile components and suspended in deionized water to obtain a precipitate, which was obtained by filtration and dried under vacuum. The dried filtrate was suspended in cold acetonitrile and filtered to obtain the final product (yield: 46.2%). NMR (400 MHz, Bruker Avance III DPX 400, DMSO-d): 7.95 (6H, dd), 7.69 (6H, m), 2.68 (2H, s), 2.34 (2H, s), 2.26 (2H, s), 2.05 (1H, s), 1.05 (9H, s). We note that reported NMR is impacted by radical-induced peak broadening. MS (MALDI-ToF) $[M + Na]^+$ m/z calculated: 628.33; $[M + H]^+$ found: 628.31. No evidence of residual compound (iii) is observed by MS.

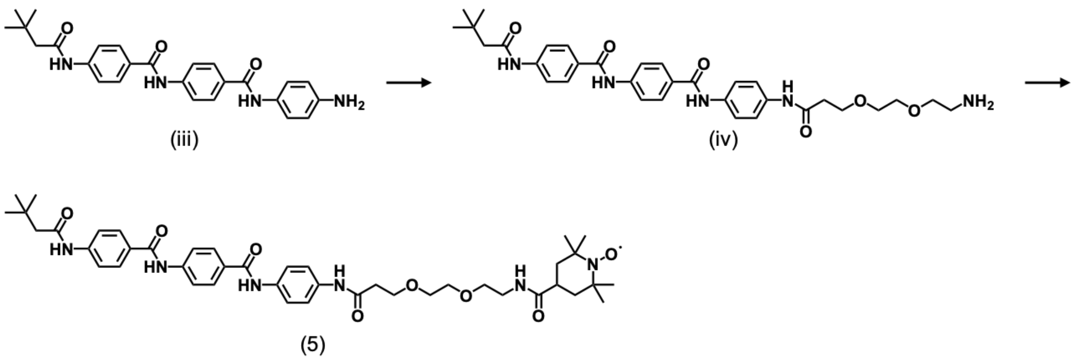

Supplementary Figure 5 | Synthesis scheme to obtain compound (**5**)

N-(2-(2-(3-((4-(4-(4-(3,3-dimethylbutanamido)benzamido)benzamido)phenyl)amino)-3-oxo propoxy)ethoxy)ethyl)-1-hydroxy-2,2,6,6-tetramethylpiperidine-4-carboxamide (**5**): A solution of compound **iv** (0.20 mmol), 4-Carboxy-TEMPO (0.40 mmol), EDC (0.60 mmol), and DMAP (0.60 mmol), and DIPEA (0.60 mmol) in dimethylformamide (4 mL) and methylene chloride (4 mL) was stirred at room temperature for 72 h. After the reaction, the solvent was removed *in vacuo*, and the remaining residue was washed with deionized water. The crude product was suspended in acetonitrile and collected by centrifugation at 4000 rpm for 3 minutes, and the precipitate was collected. The remaining product in the centrifuge tube was resuspended in solvent and centrifuged to collect three more times. All retained product was then dried under vacuum (yield: 36.5%). NMR (400 MHz, Bruker Avance III DPX 400, DMSO-*d*): 7.96 (6H, t), 7.74 (4H, m), 7.58 (2H, s), 3.73 (3H, m), 3.45 (6H, s), 2.25 (2H, s), 1.05 (9H, s) ppm. We note that reported NMR is impacted by radical-induced peak broadening. MS (MALDI-ToF) $[M + Na]^+$ m/z calculated: 809.42; $[M + Na]^+$ found: 809.45. No evidence of residual compound (iv) is observed by MS.

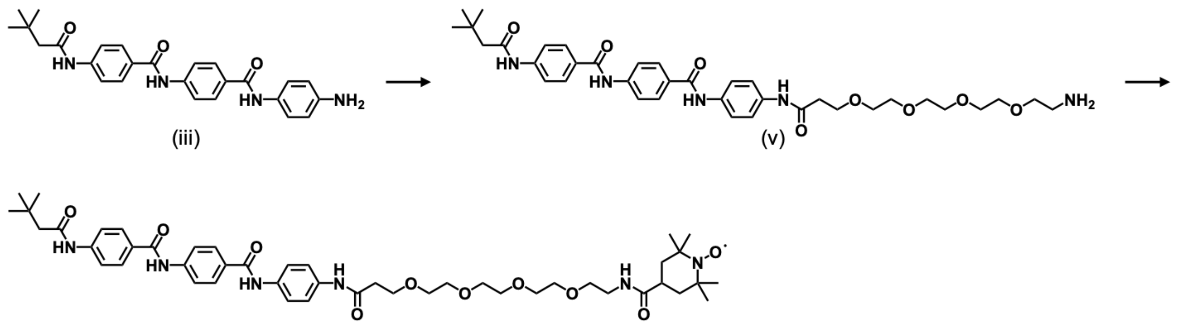

Supplementary Figure 6 | Synthesis scheme to obtain the compound **6**

N-(15-((4-(4-(4-(3,3-dimethylbutanamido)benzamido)benzamido)phenyl)amino)-15-oxo-3,6,9,12-tetraoxapentadecyl)-1-hydroxy-2,2,6,6-tetramethylpiperidine-4-carboxamide (**6**): A solution of compound (**v**) (0.25 mmol), 4-Carboxy-TEMPO (0.50 mmol), EDC (0.60 mmol), and DMAP (0.75 mmol), DIPEA (0.75 mmol) in dimethylformamide (4 mL) and methylene chloride (4 mL) was stirred at room temperature for 72 h. After the reaction, the solvent was removed *in vacuo*, and the remaining residue was washed with deionized water. The crude product was suspended in acetonitrile and collected by centrifugation at 4000 rpm for 3 minutes and the precipitate was collected. The remaining product in the centrifuge tube was resuspended in solvent and centrifuged to collect three more times. All retained product was then dried under vacuum (yield: 45.3%). NMR (400 MHz, Bruker Avance III DPX 400, DMSO-*d*): 7.96 (6H, s), 7.73 (4H, d), 7.56 (2H, s), 3.70 (2H, s), 3.52 (12H, s), 2.24 (2H, s), 1.04 (9H, s) ppm. We note that reported NMR is impacted by radical-induced peak broadening. MS (MALDI-ToF) $[M + Na]^+$ m/z calculated: 897.48; $[M + Na]^+$ found: 897.50. No evidence of residual compound (v) is observed by MS.

Infrared spectroscopy of molecular assemblies

Fourier transform infrared (FTIR) spectroscopy was conducted on suspensions of compounds (**1**) – (**3**) over the course of a 24 h sonication to produce self-assembled nanostructures. Spectra were captured using a Bruker ALPHA II spectroscope on 5 mg/mL solutions of compounds (**1**) – (**3**) in deuterated water. Solvent background was subtracted from each spectra, and deuterated water was used to minimize background signal at wavenumbers of interest.

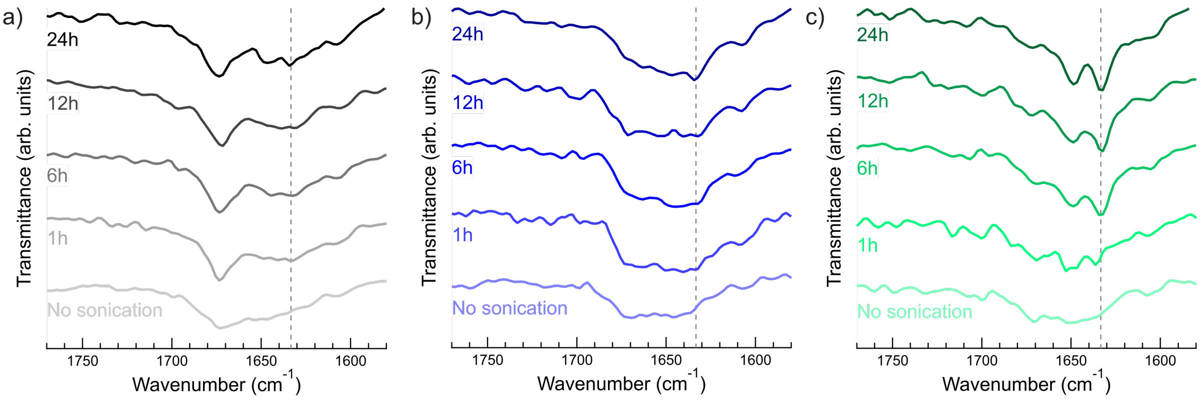

Supplementary Figure 7. FTIR of suspensions of (**a**) compound (**1**), (**b**) compound (**2**), and (**c**) compound (**3**) over the course of a 24 h bath sonication to form molecular assemblies. The emergence of a peak at 1633 cm^{-1} (dotted vertical line) corresponds to a carbonyl amide I stretch that is characteristic of β-sheet hydrogen bonding and is therefore consistent with the formation and strengthening of a hydrogen-bonding network within the molecular assemblies.

Evidence of hydrogen bonding in molecular assemblies *via* wide angle X-ray scattering

WAXS profiles of lyophilized suspensions of compound (**1**) – (**3**) assemblies were captured on a SAXSLAB instrument with a Rigaku 002 X-ray source and a DECTRIS PILATUS 300 K detector. The WAXS chamber was evacuated under dynamic vacuum prior to analysis.

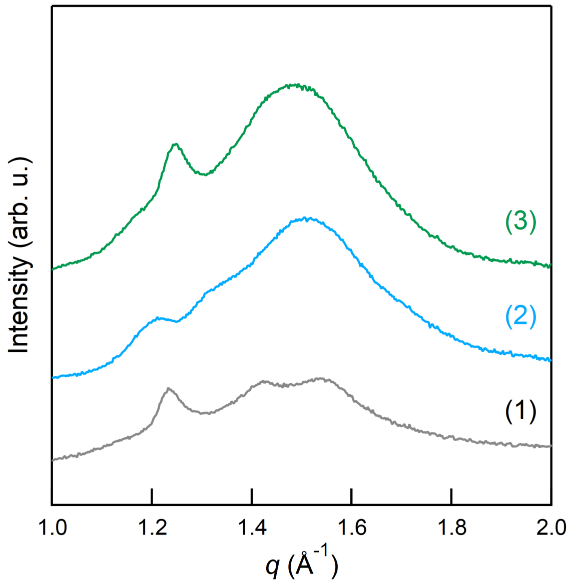

Supplementary Figure 8. Wide angle X-ray scattering (WAXS) was performed on lyophilized suspensions of compounds (**1**), (**2**), and (**3**) after self-assembly. Scattering peaks at $q \approx 1.20 - 1.25$ Å^{-1} (d ≈ 5.02 – 5.22 Å) indicate the presence of a collective hydrogen-bonding network in all nanostructures.

Site-directed spin labeling EPR of molecular assemblies

EPR spectra, especially of spin labels undergoing slow conformational motion, are prone to overfitting because of the presence of many shallow local minima in the dynamics landscapes.[1] To mitigate this, we used the Chi-Squared Cluster Analysis (CSCA) toolkit to modeling the EPR spectra reported in this manuscript. EPR spectra were fit for the parallel and perpendicular components of the axial *A*-tensor to account for spin label anisotropy, the rotational diffusion constant (D_R), and Gaussian (exchange) broadening. The selection of these fitting variables was chosen to accurately model the experimental spectra while using as few variables as possible to minimize overfitting, allowing for quantitative comparisons of the reported D_Rs. Fitting X-band EPR spectra of anisotropically rotating systems with isotropic rotational correlation times generally leads to a low-quality fit of the relative amplitudes of h(+1) and h(0) peaks.[2] However, our spectra were well-modeled by an isotropic correlation time in this respect (Figure 1), so we opted for a simpler motional model to avoid overfitting. An assumption of spherically symmetric diffusion is often consistent with experimental results for nitroxide spin labeled covalently appended into supramolecular/macromolecular ensembles.[3,4] We note that the CSCA program has been used elsewhere to accurately model macroscopic order-microscopic disorder-type systems where spin label anisotropy would be expected.[3]

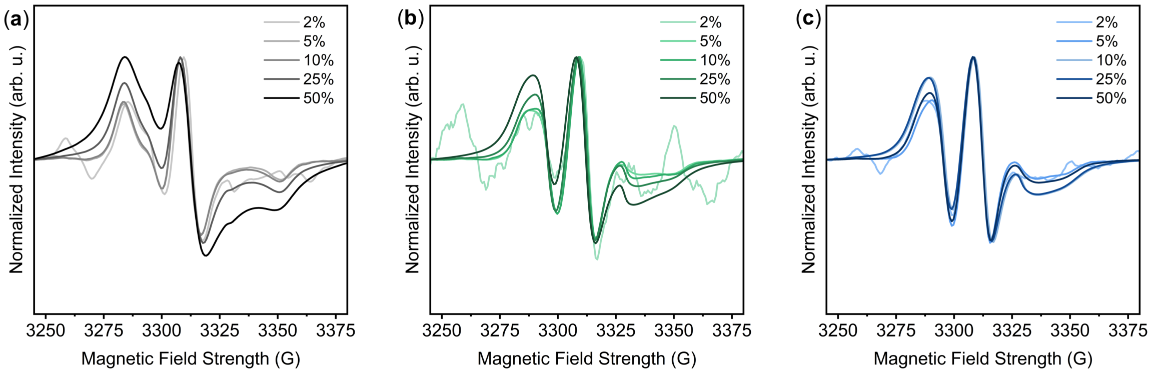

Supplementary Figure 9. Concentration series varying the mol% of spin-labeled compounds (legend) in co-assemblies with the corresponding unlabeled amphiphile. (**a**) Co-assembly of compound (**1**) with varying mol% of compound (**4**) (indicated on legend). (**b**) Co-assembly of compound (**2**) with varying mol% of compound (**5**) (indicated on legend). (**c**) Co-assembly of compound (**3**) with varying mol% of compound (**6**) (indicated on legend). Co-assemblies with 5 mol% of spin label maintain spectral features observed in 2 mol% spin-labelled co-assemblies but with significantly improved signal-to-noise. Co-assemblies with 10 mol% or more spin labelled compounds show evidence of exchange broadening. Spin labels in a 5 mol% co-assembly would be tethered to AAs whose structural domains are on average ~10 nm from one another, based on previously determined internal dimensions of AA assemblies.[5] Artifacts at low (~3260 G) and high (~3345 G) magnetic field strengths in the 2 mol% spin-labeled samples arise from Mn(II) contamination from Critoseal used to seal the EPR tubes. Intensities of all spectra are normalized to the height of the center peak (~3310 G) for comparison of broadening.

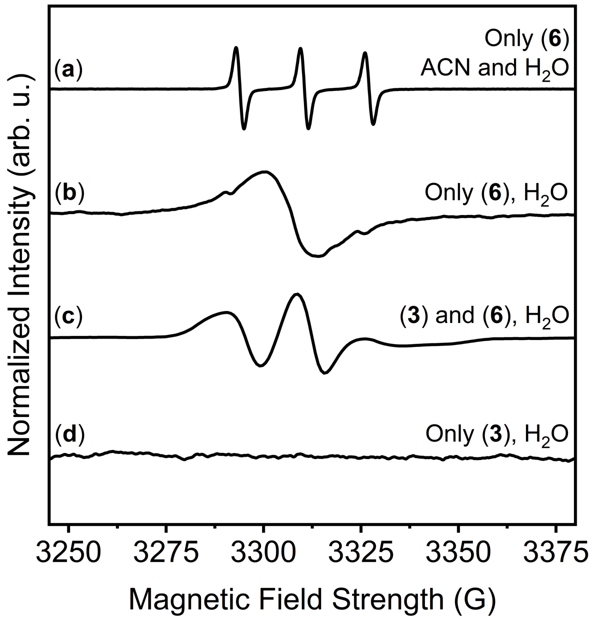

Supplementary Figure 10. EPR spectra of (**a**) a suspension of compound (**6**) in 50:50 (by vol.) acetonitrile:water; and aqueous suspensions of (**b**) compound (**6**), (**c**) 5 mol% of compound (**6**) in compound (**3**), and (d) compound (**3**). Spectras (**a**) and (**b**) contain the same molar concentration of (**6**) in solvent as the concentration of (**6**) in spectra (**c**). The suspension of compound (**6**) in acetonitrile/water shows a free-tumbling lineshape indicative of fully dissolved molecules, and the aqueous suspension of compound (**6**) demonstrates significant broadening from head group aggregation. The mixture of compound (**6**) in (**3**) exhibits a macroscopic order-microscopic disorder-like lineshape, implying co-assembly. In contrast, the aqueous suspension containing only compound (**3**) displays no EPR signal.

Small angle X-ray scattering of assemblies with Pb^{2+}

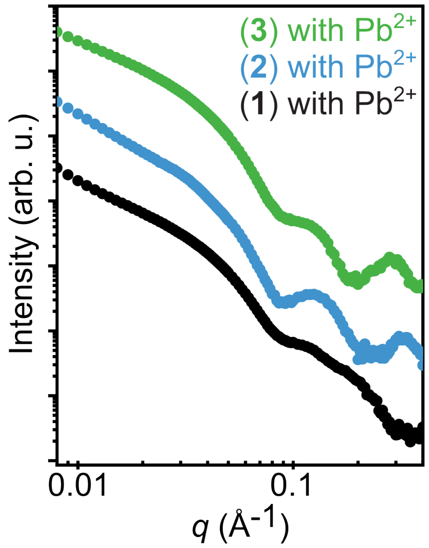

Supplementary Figure 11. SAXS of nanostructures from the self-assembly of compound (**1**), compound (**2**), and compound (**3**) after the addition of stochiometric amounts of Pb^{2+}.

Analysis of pair distance distribution functions from small angle X-ray scattering

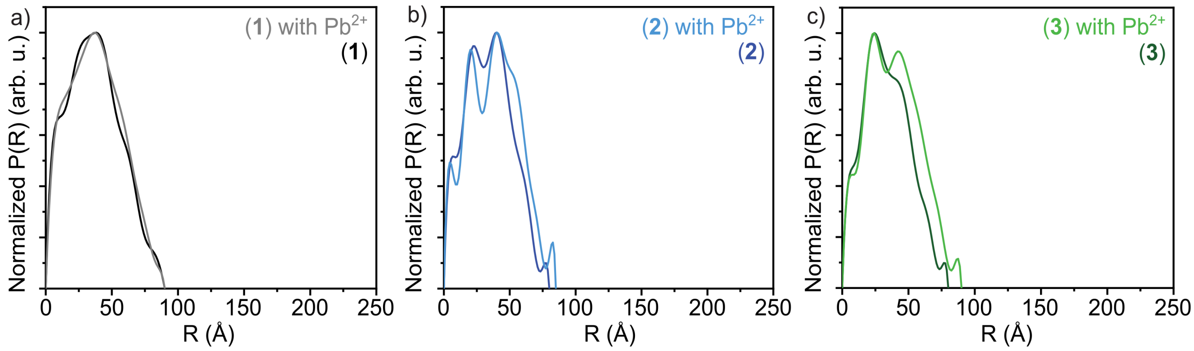

Supplementary Figure 12. GNOM PDDF fittings with low q data points removed to reduce asymmetric tailing for (**a**) compound **1**, (**b**) compound **2**, and (**c**) compound **3** nanoribbons without and with Pb^{2+}. The structural features in these fittings match those with the low q data points included (Figure 4a-c).

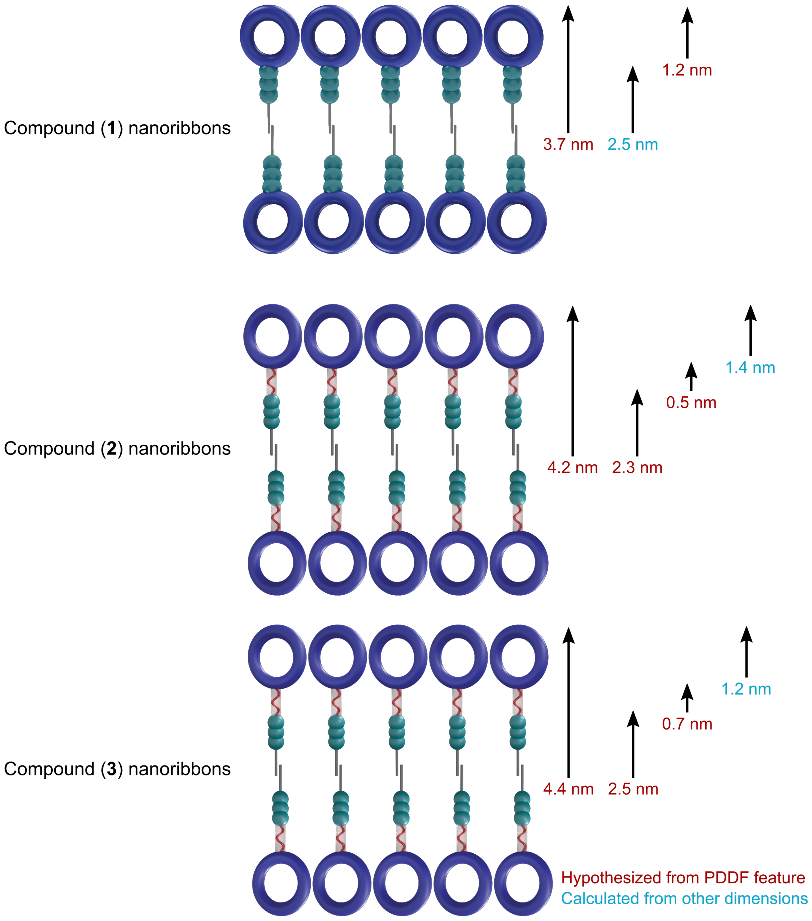

Supplementary Figure 13. Hypothesized dimensions of nanoribbon cross-sections based on observed structural features in PDDF curves.

Maintenance of nanostructures with the addition of lead (Pb^{2+})

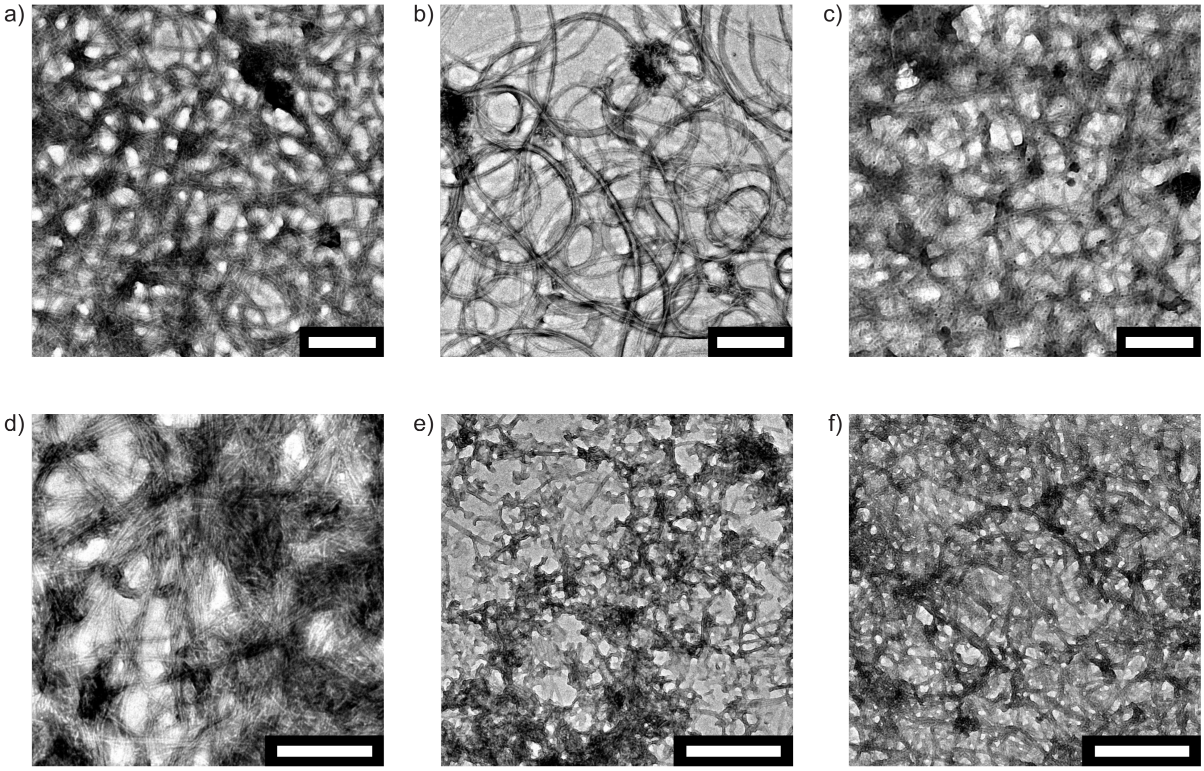

Supplementary Figure 14. TEM of nanostructures from the self-assembly of (**a**) compound (**1**), (**b**) compound (**2**), and (**c**) compound (**3**) in the absence of Pb^{2+}; and (**d**) compound (**1**), "(**e**) compound (**2**), and (**f**) compound (**3**) after the addition of stochiometric amounts of Pb^{2+}. Scale bars correspond to 300 nm.

Fits of adsorption isotherm curves to a Langmuir model

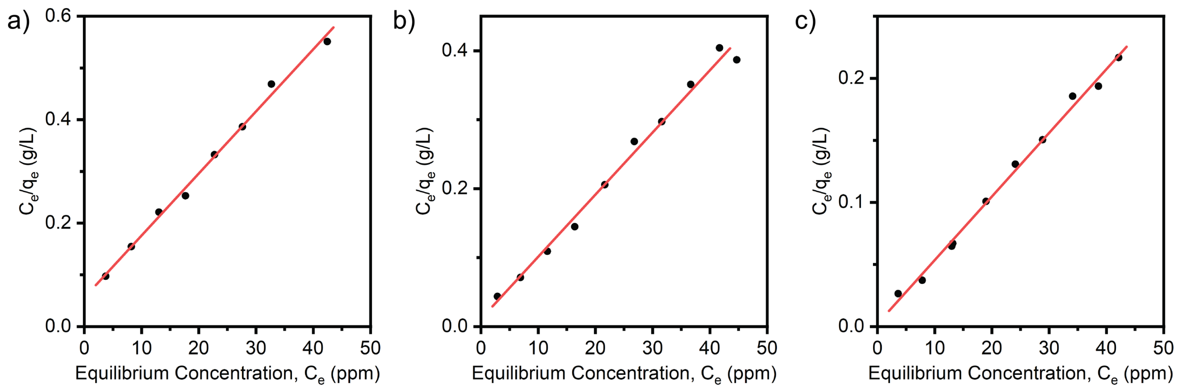

Supplementary Figure 15. Linear regression of lead adsorption isotherms for (**a**) compound (**1**), (**b**) compound (**2**), and (**c**) compound (**3**) nanoribbons, obtained by fitting equilibrium data to a Langmuir adsorption model, accompanying Figure 4g of the main text.

Supplementary References

1 Lindemann, W. R., Christoff-Tempesta, T. & Ortony, J. H. A global minimization toolkit for batch-fitting and χ^2 cluster analysis of CW-EPR spectra. *Biophys. J.* **119**, 1937-1945 (2020).

2 Etienne, E. *et al.* Guidelines for the Simulations of Nitroxide X-Band cw EPR Spectra from Site-Directed Spin Labeling Experiments Using SimLabel. *Molecules* **28**, 1348 (2023).

3 Kaser, S. J., Christoff-Tempesta, T., Uliassi, L. D., Cho, Y. & Ortony, J. H. Domain-Specific Phase Transitions in a Supramolecular Nanostructure. *J. Am. Chem. Soc.* **144**, 17841-17847 (2022).

4 Livshits, V. A. Slow anisotropic tumbling in ESR spectra of nitroxyl radicals. *J. Magn. Reson.* **24**, 307-313 (1976).

5 Christoff-Tempesta, T. *et al.* Self-assembly of aramid amphiphiles into ultra-stable nanoribbons and aligned nanoribbon threads. *Nat. Nanotechnol.* **16**, 447-454 (2021).
